# Evaluating dysphagia in Alzheimer’s disease: the significance of age and medical comorbidities,a cross-sectional study from a tertiary psychiatric hospital in Guangzhou China

**DOI:** 10.3389/fpsyt.2024.1482951

**Published:** 2024-10-08

**Authors:** Junrong Ye, Yuanxin Pan, Tingwei Zhou, Fei Liu, Yanheng Wei, Jiao Chen, Wen Wang, Xueyu Zheng, Dingjie Liu, Shengwei Wu, Zezhi Li, Jianxiong Guo, Aixiang Xiao

**Affiliations:** ^1^ The Affiliated Brain Hospital, Guangzhou Medical University, Guangzhou, China; ^2^ Key Laboratory of Neurogenetics and Channelopathies of Guangdong Province and the Ministry of Education of China, Guangzhou Medical University, Guangzhou, China; ^3^ School of Nursing, Guangzhou Medical University, Guangzhou, China; ^4^ Kiang Wu Nursing College of Macau, Macao, Macao SAR, China

**Keywords:** Alzheimer disease patients, swallowing function, influencing factors, cross-sectional study, elderly patients

## Abstract

**Objective:**

To investigate the influencing factors of dysphagia in Alzheimer’s disease (AD) patients.

**Methods:**

The study evaluated the demographic characteristics, nutritional status, social functioning, and swallowing dysfunction of 109 hospitalized AD patients.

**Results:**

The sample include 65.1% of female patients, mainly concentrate in >70 years old (72.5%). The illness duration is mainly 0~5 years (62.4%). After adjusting for confounding factors such as gender, poor lifestyle habits, illness duration, marital status, route to admission, concomitant medical illness, nutritional status, and social functioning, we find that the swallowing function in patients with AD is related to route to admission and concomitant medical illness (mainly includes: circulatory disease and respiratory disease). Age ≥90 years old and more concomitant medical illness contribute to a lower swallowing dysfunction score in patients with AD (*P*<0.05).

**Conclusion:**

Age and concomitant medical illness are the important influencing factors in swallowing dysfunction in patients with AD. Therefore, we believe that future research should focus on the treatment and care of patients with medical conditions in order to enhance the swallowing function in patients with AD.

## Highlights

- Swallowing dysfunction is a common disease in AD patients and can lead to many serious adverse consequences.
- Age and concomitant medical illness are the important influencing factors in swallowing dysfunction in patients with AD.
- Understanding the factors that influence swallowing dysfunction in patients with AD can facilitate screening the symptom in early stage and management of dysphagia to avoid serious complications.

## 1 Introduction

According to the World Health Organization, the number of people with dementia is currently 55 million, and by 2030, it is expected to increase to 78 million. The WHO global action plan on the public health response to dementia targets at least 50% of countries to diagnose 50% of the estimated number of people with dementia by 2025 (1). As the disease progresses, the self-care ability of patients with AD decreases, increasing the burden of care for families in patients with AD. Dysphagia is a geriatric syndrome that affects 10% to 30% of older adults. Oropharyngeal dysphagia was reported more commonly in older adults diagnosed as having neurologic disease (80% of AD and 60% of Parkinson disease) (2). With cerebral cortical involvement and cognitive impairment, the incidence of dysphagia in patients with severe AD is as high as 70%. Dysphagia is a common complication in patients with AD, occurring in 13%-57% and is one of the main causes of dehydration, malnutrition, aspiration pneumonia, and even asphyxia and multiple organ failure. The results of the study by Patel et al.(2018) found that patients with dysphagia had a 34% higher total hospital cost, a 2.8 times higher likelihood of needing acute follow-up care services, and a 1.7 times higher chance of dying in the hospital than patients without dysphagia, indicating that patients with dysphagia have a greater economic and survival burden.

A range of complications due to swallowing dysfunction is a major source of care burden. Consequences of dysphagia could be profound including malnutrition, volume depletion, quality of life issues, and aspiration, which may ultimately be the pivotal factor that precipitates a decline in an outcome of patients (3). In fact, dysphagia affects 1 in 25 adults annually and had significant psychosocial burden. Approximately 41% of patients had reported anxiety during mealtime and 36% avoided eating with others because of their dysphagia (4).

Understanding the factors that influence swallowing dysfunction in patients with AD can facilitate screening the symptom in early stage and management of dysphagia to avoid serious complications. This could reduce the burden of care on the patient’s family and even society. Therefore, we urgently need to find effective interventions to intervene swallowing dysfunction of patients with AD. At present, the research on the influencing factors of swallowing dysfunction mainly focuses on the age, dental condition, eating condition and course of the patients (5, 6). Our pilot observation found nutritional status, daily living skills, lifestyle habits might influence the swallowing function in patients with AD. Therefore, in this study we supplemented the assessment of the nutritional level and function of daily living in AD patients. Increasing awareness of these issues may contribute to more timely and efficient identification of Alzheimer’s disease patients with dysphagia (7), and provide scientific evidence for the development of nursing and medical interventions.

## 2 Materials and methods

### 2.1 Subjects

A consecutive sampling method was used to recruit 109 hospitalized patients with AD from February 2021 to March 2022. Inclusion criteria: 1) age≥60 years; 2) compliance with the DSM-5 criteria for Alzheimer’s disease; 3) be able to respond naturally and complete scale rating; 4) agree to sign informed consent. The World Health Organization’s classification of the elderly is as follows: 60–74 years old are considered elderly, the senile age ranges from 75 years to 90 years, and individuals aged 90 years and above are considered long-livers. Herein, the subjects were divided into three groups based on their age for analysis: 60–74 years old (group 1), 75-89 years old (group 2), and higher than 90 years old (group 3).

### 2.2 Questionnaire

Gender, age, poor lifestyle habits (drinking and smoking), illness duration, route to admission, and concomitant medical illness were collected by using a self-designed questionnaire. The scales used for evaluation include: Mini-Nutritional Assessment- Shortform (MNA-SF), Functional Questionnaire (FAQ) and Standardized Swallowing Assessment (SSA).

#### 2.2.1 Mini-nutritional assessment- shortform

The Mini-Nutritional Assessment- Shortform was simplified by Rubenstein LZ equal to 2001 based on MNA. MNA-SF contained 7 items with a total of 14 points, which was designed and validated to provide a single, rapid assessment of nutritional status in elderly patients in outpatient clinics, hospitals and nursing homes. The scale scored ≤7 as malnutrition, 8~11 as at risk of malnutrition, and ≥12 as normal nutritional status (8).

#### 2.2.2 Functional questionnaire

Functional Activities Questionnaire was developed by scholar Pfeffer et al. in 1982. The scale consisted of 10 items that mainly assess Instrumental Activities of Daily Living (IADL), including bill payment, tax administration, cooking, hobbies, following current events, traveling, remembering appointments, and taking medication. The scale used the Likert-4 scoring system, with higher scores meaning more dysfunction (9).

#### 2.2.3 Standardized swallowing assessment

Standardized Swallowing Assessment was first reported by Ellul et al. in 1996, which was scientifically designed to assess the swallowing function of patients. This scale can not only be used as an effective tool for early identification of swallowing functions, but also as a criterion for determining whether or not to remove a gastric tube. The scale was divided into 3 steps: clinical examination, water test, and normal diet test, with a total score of 18-46 (10).

### 2.3 Data collection

In the inpatient ward of a brain hospital, questionnaires were regularly distributed by four nurses trained as investigators. Once the questionnaire was completed, the nurse collected the questionnaire on the spot and checked the scale for missing or non-conformance.

### 2.4 Statistical analysis

SPSS 25.0 was applied to process data. Measurement data was presented with Mean ± SD, and counting data was expressed using frequency and percentage. The difference between the two categories was performed by independent sample t-test, one-way ANOVA for three or more categories, and non-parametric K independent sample test for rank variable; The number of influencing factors related to the number of physical diseases in AD was analyzed by linear regression analysis. Before doing multiple linear regression analysis, our study supplemented with univariate linear regression analysis to ensure the accuracy of the results. The level of significance was set at 0.05.

## 3 Results

### 3.1 The general information of participants

The majority of participants in the study were female, with the highest proportion of participants over 70 years of age. The proportion of participants with spouses is much greater than that without spouses. The majority of participants had no poor lifestyle habits. More patients were admitted to the hospital for the first time through outpatient clinics. 96.3% of participants scored < 11 in nutrition and 92.7% scored > 5 points in social functioning. (**Table 1**)

**Table 1:** General information.

Item	Frequency	Proportion (%)
Sex		
Male	38	34.9
Female	71	65.1
Age (yrs)		
60~74	52	47.7
75~89	52	47.7
≥90	5	4.6
Illness duration (yrs)		
≤5	68	62.4
5-10	22	20.2
>10	19	17.4
Marital Status		
Yes	81	74.3
No	28	25.7
Drinking		
Yes	7	6.4
No	102	93.5
Smoking		
Yes	3	2.8
No	106	97.2
First admission		
Yes	81	74.3
No	28	25.7
Route to admission		
Outpatient	75	68.8
Emergency	34	31.2
Concomitant medical illness		
Yes	96	88.1
No	13	11.9
MNA		
<11	105	96.3
≥11	4	3.7
FAQ		
≤5	8	7.3
>5	101	92.7
	**Mean**	**SD**
Swallowing function score		
SSA total	21.68	4.91
Clinical examination	8.24	1.98
Drinking water test	7.08	1.59
Normal Diet test	6.36	1.83
MMSE		
Mild dementia	6	5.5
Moderate dementia	45	41.3
Severe dementia	58	53.2
Antipsychotics		
Yes	16	14.7
No	93	85.3

The average score of the 109 participants on the swallowing function assessment was 21.68± 4.91. Among them, the average score of clinical examination was 8.24± 1.98, the average score of drinking water test was 7.08± 1.59, the average score of normal diet test was 6.36± 1.83. (**Table 1**)

### 3.2 Scores of SSA in patients with different characteristics of Alzheimer disease

According to the grouping characteristics of different variables, the independent sample t-test or one-way ANOVA was carried out, and the results showed that the analysis results of age, route to admission, concomitant medical illness, MNA score and FAQ score (*P*<<0.05) (**Table 2**).

**Table 2:** Scores of SSA in patients with different characteristics of Alzheimer disease.

Item		SSA	*t/F*	*P*
Sex				
Male		22.87 ± 5.56	1.871	0.064
Female		21.04 ± 4.44		
Age				
60~74		20.56 ± 4.20	6.352	**0.002**
74~89		22.19 ± 4.88		
≥90		28.00 ± 4.91		
Illness duration				
≤5		21.46 ± 4.78	0.424	0.655
5-10		22.55 ± 5.74		
>10		21.47 ± 4.50		
Marital Status				
Yes		21.78 ± 4.87	-0.356	0.723
No		21.39 ± 5.12		
Drinking				
No		21.75 ± 5.02	0.615	0.540
Yes		20.57 ± 2.99		
Smoking				
No		21.73 ± 4.96	0.599	0.551
Yes		20.00 ± 2.65		
First admission				
Yes		21.73 ± 4.72	0.312	0.756
No		21.43 ± 5.51		
Route to admission				
Outpatient Clinics		20.84 ± 4.01	7.431	**0.007**
Emergency		23.53 ± 6.14		
Concomitant medical illness				
Circulatory disease	Yes	22.72 ± 5.33	2.965	**0.004**
	No	20.14 ± 3.78		
Digestive disease	Yes	24.92 ± 6.52	2.477	**0.015**
	No	21.28 ± 4.56		
Respiratory disease	Yes	23.47 ± 5.80	2.938	**0.005**
	No	20.52 ± 3.85		
Urinary disease	Yes	24.45 ± 5.32	1.781	0.078
	No	21.43 ± 4.82		
Locomotor disease	Yes	20.33 ± 4.69	-0.857	0.393
	No	21.80 ± 4.94		
Endocrine disease	Yes	22.02 ± 4.98	0.643	0.522
	No	21.41 ± 4.88		
Immune disease	Yes	24.86 ± 6.94	1.788	0.077
	No	21.46 ± 4.71		
Nervous disease	Yes	22.78 ± 5.43	2.244	**0.027**
	No	20.71 ± 4.22		
Reproductive disease	Yes	23.33 ± 6.42	1.055	0.107
	No	21.53 ± 4.77		
MNA				
<11		21.80 ± 4.96	5.852	**0.000**
≥11		18.50 ± 0.58		
FAQ				
≤5		18.50 ± 1.07	-5.484	**0.000**
>5		21.93 ± 5.01		
MMSE				
Mild dementia		20.50 ± 2.31	0.462	0.631
Moderate dementia		21.33 ± 4.69		
Severe dementia		22.07 ± 5.06		
Antipsychotics				
Yes		21.69 ± 6.12	0.008	0.994
No		21.68 ± 4.71		

Bold highlight those value of P<0.005, they are statistical significant.

### 3.3 Uni-variate linear regression

The above statistically significant results were analyzed by univariate linear regression, and the results showed that age (≥90 years old), route to admission (emergency), concomitant medical illness, and FAQ score (>5) (*P*< 0.05). (**Table 3**)

**Table 3:** Uni-variate linear regression.

Item	*β*	*P*
Age		
60~74	-0.219	**0.022**
75~89	0.100	0.299
≥90	0.283	**0.003**
Route to admission		
Emergency	0.255	**0.007**
Outpatient	-0.255	**0.007**
Concomitant medical illness		
Circulatory disease	0.260	**0.006**
Digestive disease	0.233	**0.015**
Respiratory disease	0.295	**0.002**
Nervous disease	0.212	**0.027**
MNA		
<11	0.127	0.189
FAQ		
>5	1.970	**0.057**

Bold highlight those value of P<0.005, they are statistical significant.

### 3.4 Linear regression analysis of factors influencing Swallowing function

The linear regression analysis model was established by taking swallowing function scores in patients with Alzheimer disease as the dependent variable, and the statistically significant variables of univariate linear regression as independent variables. The results showed that age (≥90 years old), concomitant medical illness (circulatory disease and respiratory disease)(*P*<0.05). (**Table 4**)

**Table 4:** Linear regression analysis of factors influencing Swallowing function.

	Non-standardized	Coefficients	standardized coefficients	*t*	*P*	*95% CI*
	β	Standardized Error	Beta			
(Constant)	15.544	1.636	–	9.504	***P*<0.001**	12.301
Age 60~74	-0.89			-0.968	0.335	
Age ≥90	5.502	2.041	0.235	2.695	**0.008**	1.454, 9.550
Emergency	0.154			1.719	0.089	
Outpatient	-0.154			-1.719	0.089	
Circulatory disease	2.793	0.870	0.279	3.209	**0.006**	1.067, 4.519
Digestive disease	0.145			1.638	0.104	
Respiratory disease	2.442	0.867	0.245	2.442	**0.002**	0.722, 4.161
Nervous disease	0.112			1.257	0.211	
FAQ >5	0.153			1.776	0.079	

Bold highlight those value of P<0.005, they are statistical significant.

## 4 Discussion

Previous studies had found that patients with AD had a higher risk of dysphagia, but some clinical features and associated factors remained unclear (11). On the basis of empirical findings, this study investigated the swallowing function of patients with AD and reported that swallowing dysfunction was related to the age and its concomitant medical illness, including circulatory disease and respiratory disease.

Our study found that circulatory and respiratory diseases were important factors in swallowing dysfunction in patients with AD. Circulatory system dysfunction, such as reduced blood circulation or insufficient blood supply, might affect swallowing function. Intracranial circulation disorders could cause ischemia due to vascular wall lesions, blood composition or hemodynamic changes, including sensory dysfunction, autonomic nerve dysfunction, and motor nerve dysfunction, resulting in dysphagia (12). Insufficient blood supply to brain lead to impaired nerve function that regulates swallowing movement, resulting in dysphagia. Stroke is one of prevalent circulatory system diseases which influences the progression of dysphagia, because the lack of blood supply to the brain would affect function of nerves that control swallowing organ (13). In addition, studies found that long-term hypertension would affect blood circulation via hardening of brain vessels, abnormal brain circulation causes the injury of pharyngeal cortex center, leading to the descended cortical fibers, bulbar swallowing center and extrapyramidal system (14, 15). Consequently, prolonged oral transit time, delayed pharyngeal swallowing, and even aspiration during swallowing procedure, might occur after stroke (16).

This investigation suggested dysphagia was associated with respiratory system diseases, for instance chronic obstructive pulmonary disease (COPD), and pneumonia. Patients with respiratory diseases experience general respiratory symptoms that disrupt eating behavior, leading to choking episodes. This might due to the abnormal cough reflex cause food or liquids to stray into the airway and trigger dysphagia (17). Respiration and swallowing movement share reflective neuroanatomical mechanisms and pathways, individuals with tachypnea demonstrates shortened duration of apnea, disrupted swallowing, and even oropharyngeal dysphagia (18). In addition, long-term breathing difficulties and throat discomfort might induce dysphagia because of pharyngeal muscles fatigue (19). Thus, additional attention should be paid to medical comorbidities when screening swallowing function in patients with AD.

Aging was a vital factor influencing swallowing function in patients with AD: degenerative brain nerve function and abnormal swallowing reflex function weakened transport capacity and coordination of oral muscle groups, therefore older adults had greater risk of dysphagia (20). Research by Mayla Izumi et al. (2018) highlighted damaged tongue pressure had impacts on insufficient saliva secretion, weakened immunity, and impaired tongue motor function, resulting in dysphagia in aged adults (21). Given that aged patients with AD had clinical risk of dysphagia, interventions targeting on swallowing function should be conducted in early stage.

Previous study by Park, Y.H., et al. (2013) showed patients with moderate to severe dementia were at risk of dysphagia (22). In our study, the SSA scores (mean ± s.d.) of patients with mild (5.5%), moderate (41.3%), and severe (53.2%) AD were 20.50 ± 2.31, 21.33 ± 4.69, 22.07 ± 5.06, respectively. We had noticed an incline of SSA score with the increasing severity of AD, but further analysis had not clarified statistical different among three groups. These might due to proportions of participants with different dysphagia level varied significantly. To note, clinical evidence indicated that long-term use of antipsychotic medication lead to tardive dyskinesia, a movement disorder that disrupts effective swallowing (23, 24). In our study, we recorded the application of antipsychotics, but did not indicate significant difference in SSA score between participants underwent antipsychotics therapy or not. These might due to that clinically only very low does of antipsychotics was administrated to alleviated behavioral and psychological symptoms of dementia (BPSD), particularly in patients of first admission (74.3% in our study). Furthermore, it is worthy to mention the finding by Marta Miarons (2018) that the role of decreased physical condition caused by aging process and AD had greater impact on directing dysphagia than that of antipsychotics treatment (25). Therefore, future study unveiling how and to what extent antipsychotics influence dysphagia is demanding.

In summary, dysphagia of patients with AD is affected by age and medical comorbidities, despite the number of patients with AD is growing annually, understanding the caring demand benefits raising public awareness and developing targeted intervention (26). Limitation in this study should be mentioned: participants were recruited from a public psychiatric hospital in Guangzhou, China, this might affect the generality of our findings; besides, further study should focus on other factors encompassing AD combined with dysphagia, particularly gender balance, edentulism and frailty.

## Data Availability

The original contributions presented in the study are included in the article/supplementary material. Further inquiries can be directed to the corresponding authors.
